# Combined endoscopic and radiologic intervention for management of acute perforated peptic ulcer: a randomized controlled trial

**DOI:** 10.1186/s13017-022-00429-9

**Published:** 2022-05-24

**Authors:** Said Negm, Hatem Mohamed, Ahmed Shafiq, Taha AbdelKader, Adel Ismail, Mahmoud Yassin, Bassam Mousa, Mohamed Abozaid, Yasser A. Orban, Mazoun Al Alawi, Ahmed Farag

**Affiliations:** 1 Faculty of Medicine, Zagazig University, Zagazig, Egypt; 2 Sur Hospital, Ministry of Health, Muscat, Oman; 3 Ismailia Teaching Oncology Hospital, Ismailia, Egypt; 4 Ministry of Health, Muscat, Oman

**Keywords:** Peptic ulcer, Stent, Clipping, Surgical emergency, Endoscopy

## Abstract

**Background:**

Peptic ulcer perforation is a common life-threatening surgical emergency. Graham omental patch is performed for plugging of perforated peptic ulcer. Many endoscopic methods have been used to treat acute perforated peptic ulcer such as over the scope clips, standard endoscopic clips, endoscopic sewing and metallic stents. The main idea in endoscopic management of acute perforated peptic ulcer is early decontamination and decrease sepsis by interventional radiologic drainage.

**Methods:**

This is a prospective randomized controlled clinical trial. This study included patients who were developed acute perforated peptic ulcer manifestations and were admitted to our hospital between December 2019 and August 2021. Sample size was 100 patients divided into 2 equal groups. Endoscopic group (EG): included 50 patients who were subjected to endoscopic management. Surgical group (SG): included 50 patients who were subjected to surgical management.

**Results:**

One hundred patients were randomized into 2 groups: SG (50) and EG (50). Median age of patients was 36 (range 27:54) and 47 (range 41:50) years-old in SG and EG, respectively. Males constituted 72% and 66% in SG and EG, respectively. Median length of postoperative hospital stay was 1 (range: 1–2) days in EG, while in SG was 7 (range 6–8) days. Postoperative complications in SG patients were 58% in form of fever, pneumonia, leak, abdominal abscess, renal failure and incisional hernia (11%, 5%, 5%, 3%, 2% and 3%, respectively). Postoperative complications in EG patients were 24% in form of fever, pneumonia, leak, abdominal abscess, renal failure and incisional hernia (10%, 0%, 2%, 0%, 0% and 0%, respectively).

**Conclusion:**

Combined endoscopic and interventional radiological drainage can effectively manage acute perforated peptic ulcer without the need for general anesthesia, with short operative time, in high risk surgical patients with low incidence of morbidity & mortality.

## Introduction

Peptic ulcer perforation is a common life-threatening surgical emergency [1]. Perforated peptic ulcer requires either laparoscopic or open surgical repair and is associated with high morbidity (35%) and mortality (5–16%) rates [2]. The most common surgery for perforated peptic ulcer is the use of Graham patch or oversewing the ulcer. Graham patch technique was described by Cellan-Jones in 1929 [3] and Graham in 1937 [4]. Conservatively, high risk surgical patients who cannot undergo definitive surgical repair may be managed with either Taylor’s method [5] or percutaneous drainage. Taylor’s methods consists of nasogastric tube decompression [5], and is associated with high mortality rate. In comparison with Taylor’s method, percutaneous drainage reduced the mortality rate by 20% [6]. Endoscopic techniques for management of perforated peptic ulcer have been gaining popularity using over-the-scope or standard clips, endoscopic sewing and metallic stents [7]. The main idea in endoscopic management of acute perforated peptic ulcer is early decontamination and decrease sepsis by interventional radiologic drainage [8].

## Objectives

To assess the effectiveness of concurrent endoscopic and interventional radiological intervention versus surgical (open/laparoscopic) intervention in the management of acute perforated peptic ulcer in reducing morbidity and mortality rates, and overall surgical complications**.**

## Patients and methods

### Patients

This prospective randomized controlled clinical trial included all patients who developed the manifestations of acute perforated peptic ulcer and referred to the Zagazig University Hospital Emergency Department between December 2019 and August 2021. The study was prospectively approved by Zagazig University Faculty of Medicine Institutional Review Board (Approval Number: 11195/20.12.2019) and was retrospectively submitted in clinicaltrials.gov in September 2021 (NCT05051683). The study was performed in accordance with the code of ethics of the World Medical Association (Declaration of Helsinki) for studies involving human subjects. Written informed consent was obtained from all participants after explaining to them all the study procedures with its benefits and hazards. Patients ≥ 18- ≤ 60-years-old, with acute perforated gastric or duodenal ulcers, with early chemical peritonitis and no septic shock were deemed eligible for randomization. We excluded patients who were < 18- > 60-years-old, in severe septic shock, or presence of massive amount of intraperitoneal, subhepatic, perisplenic and pelvic free fluid. Another procedural exclusion was the size of perforation > 30 mm in diameter. The Duration from the time of presentation to intervention ranges from 12–36 h, provided that patients did not develop septic shock.

Included eligible patients were simply randomized at a 1:1 ratio to “Surgery Group (SG)” or “Endoscopic Group (EG)” regardless of the site of the peptic ulcer via the drawing of sealed envelopes containing computer-generated random numbers prepared by a third party before the start of the intervention.

The sample size was calculated by using open Epi program depending on the following data; confidence interval 95%, power of the test 80%, ratio of unexposed/exposed 1, percent of patient with leak with surgical technique 12%, and those with leak with endoscopy 0.14% [9], odds ratio 0.21 and risk ratio 0.25.

Primary and secondary outcomes were leak and mortality in each group after the intervention during the 3-month follow-up period, respectively.

### Diagnosis

After full history taking and complete physical examination, acute perforated peptic ulcer was clinically suspected and then confirmed by laboratory investigations (complete blood picture, liver and kidney functions, coagulation profile), radiological imaging (plain erect chest X-ray abdomen, abdominal US to know amount of contamination, CT abdomen with oral and I.V contrast to know site of perforation).

### Intervention

For patients in EG, we began with assessment of the site & size of perforation. In case of the perforated duodenal ulcer (due to narrow lumen), endoscopic stent (fully covered self-expanded metallic stent, FCSEMS) was deployed. In case of gastric ulcer (due to capacious cavity) with a small diameter of perforation (< 10 mm), FCSEMS and Over-The-Scope Clipping (OTSC, Ovesco Endoscopy AG, Tubingen, Germany) were performed, while in gastric ulcer with a wide diameter of perforation (20–30 mm), FCSEMS and endoscopic suturing (OverStitch (36), Apollo Endo-surgery, TX, United States) were utilized. Concurrently, the interventional radiology team subcutaneously drained the intraperitoneal free fluid using 2 intra-peritoneal tubes that were placed under US guidance in the sub-hepatic region and in the pelvis.

For SG patients, either open or laparoscopic surgical exploration, and primary repair of perforation supported by Graham's omental patch were performed. Two intra-peritoneal tube drains were placed in the sub-hepatic region, and in the pelvis.

On the first postoperative day (POD 1), all patients in both groups underwent methylene blue test and the result was considered positive if the blue color appeared in the drain.

During the hospital day, all patients in both groups underwent periodic clinical examination and laboratory tests. In case of suspected leak post repair, abdominal CT scan (with oral and I.V contrast) and upper GI endoscopy were performed. Patients were followed-up for at least 3-months post repair.

### Statistical analysis

Analysis of data was performed using SPSS (Statistical Package of Social Services) version 22. Quantitative variables were described as mean (± SD, standard deviation) and median (range) according to Shapiro test of normality. Qualitative variables were described as a number and a percent. Chi-square test was used to compare qualitative variables between the 2 groups. Fisher exact test was used when one expected cell or more are less than 5. Unpaired t-test was used to compare quantitative variables, in parametric data (SD < 30% of the mean). Mann–Whitney test was used instead of unpaired t-test in nonparametric data (SD > 30% of the mean). The results were considered statistically significant when the significant probability was less than 0.05 (P < 0.05). P-value < 0.001 was considered highly statistically significant (HS), and *P* value ≥ 0.05 was considered statistically insignificant (NS) [10].

## Results

**Fig. 1:**
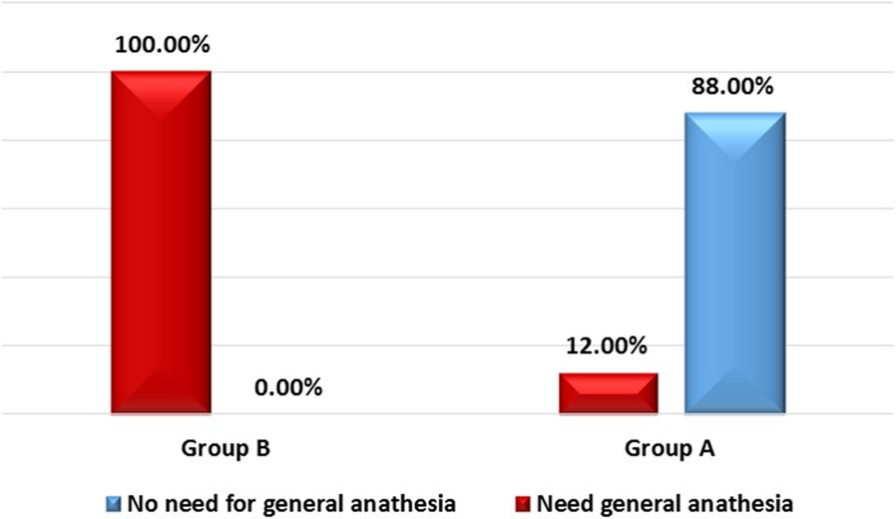
Need for general anesthesia in group A ( EG) & group B ( SG)

**Fig. 2:**
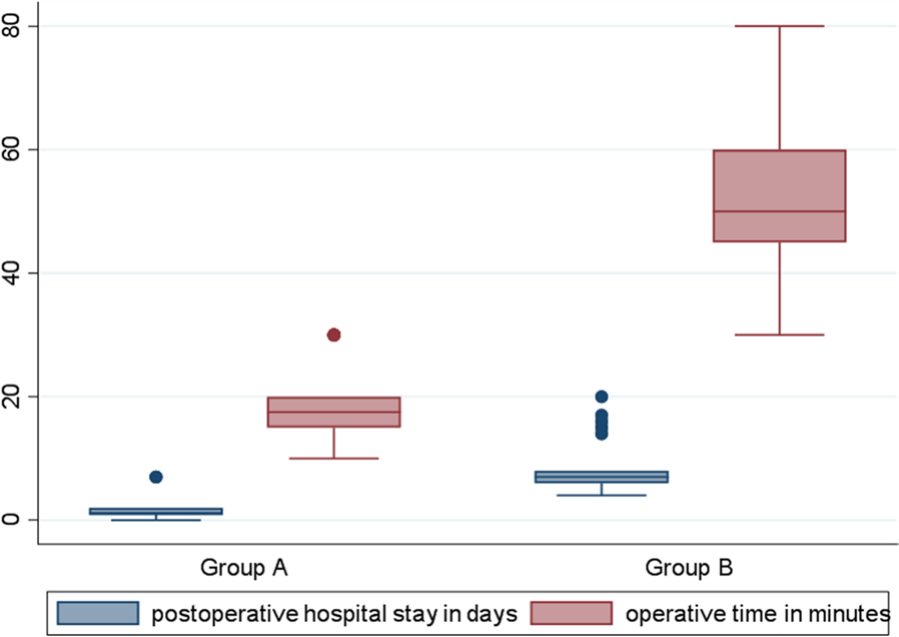
Box plot to compare operative time and hospital stay between group A (EG) Group B (SG)

**Fig. 3:**
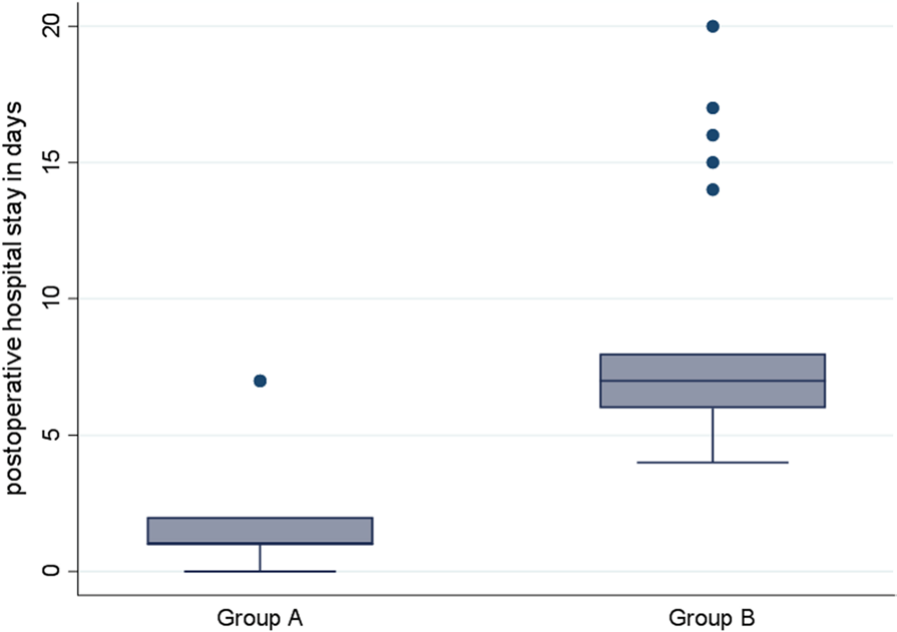
Box plot to compare operative time between Groups A (EG) and B (SG)

**Fig. 4:**
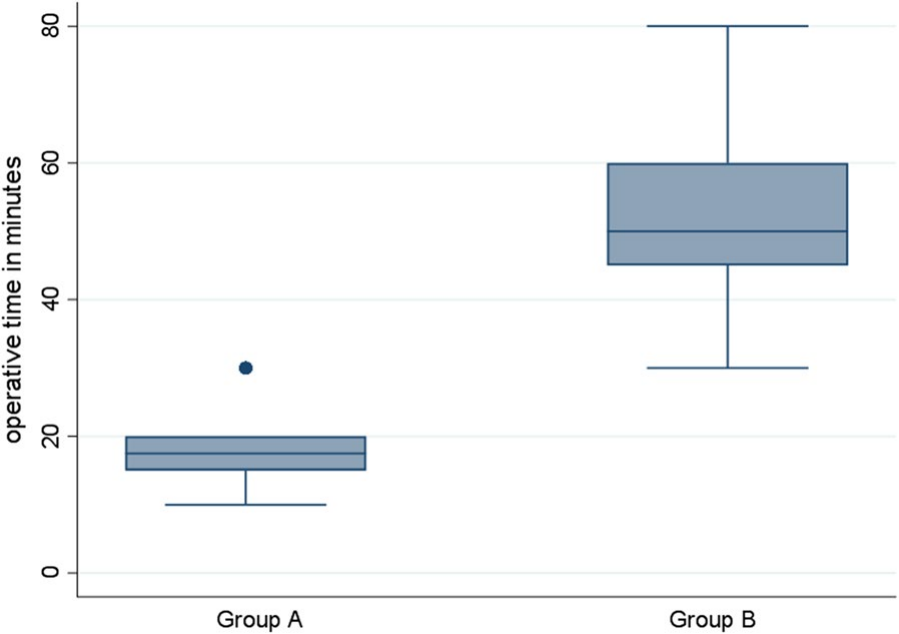
Box plot to compare operative time between groups A (EG) and B (SG)

Of 158 patients who presented with manifestations of acute perforated peptic ulcer, eligible 100 patients were randomized into 2 groups: 50 in SG and 50 in EG as in Tables 1,2,3 and 4. Median age of patients was 47 (range: 41–50) and 36 (range: 27–54) years-old in EG and SG, respectively. Males constituted 66% (33/50) and 72% (36/50) of patients in EG and SG, respectively. All patients in SG (Fig. 1) and only 6 (12%) patients in EG underwent general anesthesia. Perforated gastric ulcer was reported in 21 and 15 patients in EG and SG, respectively, while perforated duodenal ulcer was reported in 29 and 35 in EG and SG, respectively. In SG, 10 patients underwent open surgical exploration and primary repair of perforated peptic ulcer (gastric ulcer in 5 and duodenal ulcer in 5 patients), while the remaining SG patients underwent laparoscopic repair of perforated peptic ulcer. Median operative time (Figs. 2, 3, 4) was 17.5 (range: 15–20) and 50 (range: 45–60) minutes in EG and SG, respectively. The methylene blue test was negative in 48 (96%) & 45 (90%) patients in EG and SG, respectively.

**Table 1:** Characteristics of the patients

	N	%
*Sex*		
Male	69	69
Female	31	31
*Blue dye test postoperative*		
Negative	93	93
Positive	7	7
*Need for general anesthesia*		
No	44	44
Yes	56	56
*Success to close the perforation*		
No	7	7
Yes	93	93
*Mortality*		
No	97	97
Yes	3	3
*Post-operative decrease in CRP & WBC count*		
No	7	7
Yes	93	93
*Postoperative complication*		
No	59	59
Yes	41	41
*Postoperative complications*		
No complications	59	59
Fever	21	21
Pneumonia	5	5
Leak	7	7
Abdominal abscess	3	3
Renal failure& incisional hernia	2	2
Incisional hernia	3	3

**Table 2:** Operative time and postoperative hospital stay

	Mean ± SD	Median(IQR)	Maximum/Minimum
Age (years)	43.86 ± 14.46	44(34:50)	85:20
Operative time in minutes	35.68 ± 19.11	30(17.5:50)	80:10
Postoperative hospital stay (days)	4.45 ± 3.89	5(1:7)	20:0

**Table 3:** Chi-square test used to compare groups *Exact test correction

	EG	SG	*P* value
	N(%)	N(%)	
*Sex*			
Male	33(66)	36(72)	0.517
Female	17(34)	14(28)	
*Blue dye test postoperative*			
Negative	48(96)	45(90)	*0.436
Positive	2(4)	5(10)	
*Need for general anesthesia*			
No	44(88)	0(0)	< 0.001
Yes	6(12)	50(100)	
*Success to close the perforation*			
No	2(4)	5(10)	*0.436
Yes	48(96)	45(90)	
*Mortality*			
No	50(100)	47(94)	*0.242
Yes	0(0)	3(6)	
*Post-operative decrease in CRP & WBC count*			
No	2(4)	5(10)	*0.436
Yes	48(96)	45(90)	
*Postoperative complication*			
No	38(76)	21(42)	0.001
Yes	12(24)	29(58)	
*Postoperative complications *			
No complications	38(76)	21(42)	0.001
Fever	10(20)	11(22)	0.806
Pneumonia	0(0)	5(10)	*0.056
Leak	2(4)	5(10)	*0.436
Abdominal abscess	0(0)	3(6)	*0.242
Renal failure& incisional hernia	0(0)	2(4)	*0.495
Incisional hernia	0(0)	3(6)	*0.242

**Table 4:** Mann–Whitney U test used to compare groups

	EG	SG	*P* value
	Median(IQR)	Median(IQR)	
Age (years)	47(41:50)	36(27:54)	0.014
Operative time (minutes)	17.5(15:20)	50(45:60)	< 0.001
Postoperative hospital stay (days)	1(1:2)	7(6:8)	< 0.001

**Fig. 5:**
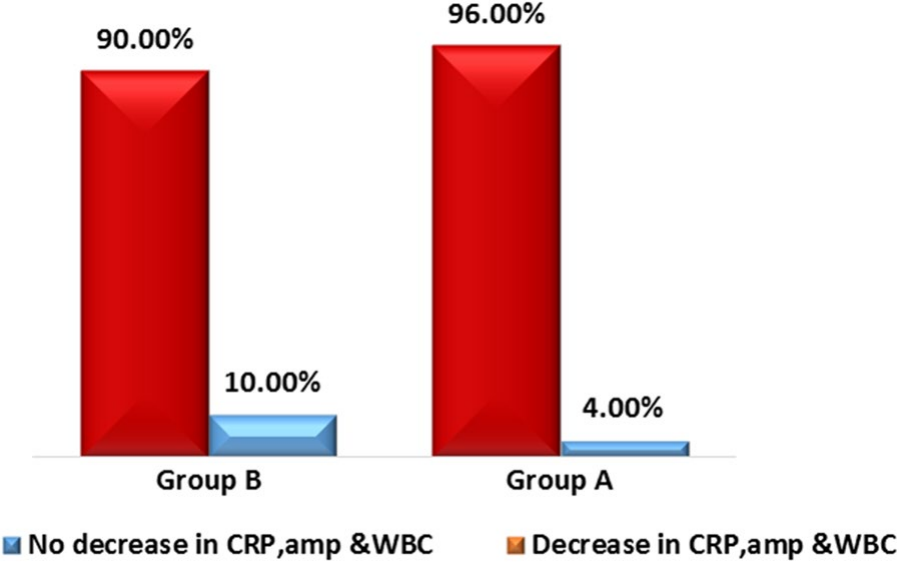
Decrease in CRP & WBCs in group A(EG) & group B ( SG)

The observed incidence of success to close the perforation was 96% (48/50) and 90% (45/50) in EG and SG, respectively. The median length of hospital stay (Figs. 2, 3, 4) was 1 (range: 1–2) and 7 (range: 6–8) days in EG and SG, respectively. C-reactive protein and white blood cells count decreased (Fig. 5) in 48 (96%) and 45 (90%) patients in EG and SG, respectively. The observed incidence of postoperative complications was 58% in SG compared with 24% in EG. The observed incidence of complication-type specific in SG was fever 11%, pneumonia 5%, leak 5%, abdominal abscess 3%, renal failure 2% and incisional hernia 3%. The observed incidence of complication-type specific in EG was fever 10% and leak 2%, while no patients in EG developed pneumonia, abdominal abscess, renal failure or incisional hernia. Mortality rate was 6% (3/50) in SG, while no patients died in EG. In multivariable linear regression model, the significant predictors of increased length of hospital stay were increased age in years (*P* = 0.018), assignment to the surgical intervention group (*P* < 0.001, highest predictor), prolonged operative time (*P* = 0.05) and blue dye test (*P* < 0.001).

## Discussion

Combined endoscopic and interventional radiology approach has become a promising tool in the management of acute perforated peptic ulcer. The use of the endoscopy offers different modalities such as stenting, clipping and endo-suturing. In addition, interventional radiology is being used for drainage of the intraperitoneal collection at the same session. In this study, the endoscopic intervention demonstrated reduced need for using of general anesthesia in most of the cases, short operative time, short postoperative hospital stay, decreased incidence of postoperative complications (no incisional hernia or seroma), early tolerance of oral feeding, no surgical incisions, and can be used in high risk surgical patients with high success rate.

In this study, OTSC was used in 15 patients (30%) with only perforated gastric ulcer, without clip migration or development of post-OTSC stricture during the 3-month follow-up period. We started deploying the clips perpendicular to the long axis of the defect. If needed, more than one clip was sequentially deployed, starting at edge of the defect towards the center. Standard clips were passed through-the-scope to achieve superficial tissue apposition engaging the mucosa and submucosa (with 1.2-mm-wide and 6-mm-long arms capable of an approximately 12-mm grasp) and were used in conjunction with thermal ablation or mechanical scraping of the tissue around the edges of the defect to achieve a more resilient seal. OTSC demonstrated a statistically significant successful closure rate for GI perforations and leaks (average 82%) compared to that of fistulas (42.9%), and long-term success of OTSC as a primary than a rescue therapeutic option (69% vs. 46.9%, respectively,* P *= 0.004) for managing GI perforations and leaks, as well [11]. A systematic review concluded that OTS clips achieved successful closure rate of 51.5% in GI fistulae and 66% in GI anastomotic leaks [12]. OTS clips were used to close narrow perforation usually less than 1 cm and in cases of gastric ulcer perforations only as the stomach has a capacious lumen.

OverStitching is theoretically an optimum method of site perforation closure in gastric ulcer perforation only (not duodenal ulcer) because it is the only true full thickness site perforation closure and performed endoscopically despite being a complex procedure. In this study, OverStitching was used in 5 patients (10%) who under endoscopic management. The procedure began with de-epithelialization of the edges of the site perforation using diathermy cautery before applying the OverStitching system. We did not experience post-OverStitch gastric leak. It is usually used in gastric ulcer perforation with size 1–2 cm.

In this study, we used a fully covered stent in 30 patients (60%) (Mega stent, Taewoong Medical Industries, Gyeonggi-do, South Korea) ultra-large and long (length: 24 cm, diameter: 36 mm) stent in both gastric & duodenal ulcer perforations. We did not experience any complication with Mega stent, particularly migration, thanks to the design of Mega stent that fits well with reduction in migration. It completely covers the whole perforation site and its lower end rests in second part of the duodenum. The reported migration incidence of FCSEMS is twice that of partially covered stents (26% vs. 13%) [13]. Of note, between the groups, EG showed statistically significant shorter length of stay (< 0.001). Surprisingly, the postoperative complications in SG such as pneumonia, abdominal collection and renal failure were not predicators of the length of hospital stay in this group of patients.

This study has some limitations. The small sample size that may not give powerful statistical conclusions. Exclusion of patients with age below 18 years old is another limitation. Regarding the de-epithelization of the edges of fistula either for OTSC or OverStitch, there was not a single method, and it was up to the endoscopist to use argon plasma laser or mechanical scrapping of the edges. Moreover, this study showed only 3-month follow-up period. The strength of the present study is being a randomized controlled trial and comparing different endoscopic interventions on one hand with the surgical intervention on the other hand.

## Conclusion

Combined endoscopic & interventional radiology approach can effectively manage acute perforated peptic ulcer without need for general anesthesia, with short operative time and length of hospital stay, in high risk surgical patients with low incidence of morbidity & mortality.

## Data Availability

All data generated during this study are included in this published article and its supplementary information files. Further minor datasets are available from the corresponding author on reasonable request.

## Abbreviations

Ppu: Perforated peptic ulcer
EG: Endoscopic group
SG: Surgical group
FCSEMS: Fully covered self-expanded metallic stent
OTSC: Over-the-scope clipping
